# SplicePie: a novel analytical approach for the detection of alternative, non-sequential and recursive splicing

**DOI:** 10.1093/nar/gkv242

**Published:** 2015-03-23

**Authors:** Irina Pulyakhina, Isabella Gazzoli, Peter A.C. ’t Hoen, Nisha Verwey, Johan den Dunnen, Annemieke Aartsma-Rus, Jeroen F.J. Laros

**Affiliations:** 1 Department of Human Genetics, Leiden University Medical Center, Leiden, The Netherlands; 2 Leiden Genome Technology Center, Leiden University Medical Center, Leiden, The Netherlands

## Abstract

Alternative splicing is a powerful mechanism present in eukaryotic cells to obtain a wide range of transcripts and protein isoforms from a relatively small number of genes. The mechanisms regulating (alternative) splicing and the paradigm of consecutive splicing have recently been challenged, especially for genes with a large number of introns. RNA-Seq, a powerful technology using deep sequencing in order to determine transcript structure and expression levels, is usually performed on mature mRNA, therefore not allowing detailed analysis of splicing progression. Sequencing pre-mRNA at different stages of splicing potentially provides insight into mRNA maturation. Although the number of tools that analyze total and cytoplasmic RNA in order to elucidate the transcriptome composition is rapidly growing, there are no tools specifically designed for the analysis of nuclear RNA (which contains mixtures of pre- and mature mRNA). We developed dedicated algorithms to investigate the splicing process. In this paper, we present a new classification of RNA-Seq reads based on three major stages of splicing: pre-, intermediate- and post-splicing. Applying this novel classification we demonstrate the possibility to analyze the order of splicing. Furthermore, we uncover the potential to investigate the multi-step nature of splicing, assessing various types of recursive splicing events. We provide the data that gives biological insight into the order of splicing, show that non-sequential splicing of certain introns is reproducible and coinciding in multiple cell lines. We validated our observations with independent experimental technologies and showed the reliability of our method. The pipeline, named *SplicePie*, is freely available at: https://github.com/pulyakhina/splicing_analysis_pipeline. The example data can be found at: https://barmsijs.lumc.nl/HG/irina/example_data.tar.gz.

## INTRODUCTION

During messenger RNA (mRNA) splicing, introns are removed and exons are joined to generate a mature mRNA or a transcript. One transcript, usually chosen arbitrarily, to which all other transcripts are compared is called the ‘reference transcript’. The deviations from this standard reference transcript are called alternative transcripts.

Data obtained with recent technologies, such as next-generation sequencing (NGS) of a whole transcriptome, revealed that around 90% of human genes undergo alternative splicing (1,2). Alternative splicing of a gene comes in different flavours, such as skipping (a part of) an exon (3) or intron retention (4). The most prominent event is exon exclusion (or skipping), which has been predicted to occur in at least two-thirds of all human genes (based microarray profiling and expressed sequence tags (EST)–cDNA sequence data (5)).

Splicing generally occurs co-transcriptionally (6,7), and alternative splicing can be influenced by the speed of transcription (8,9). It has been reported that splicing does not always progress sequentially from the 5′ to the 3′ end of a gene and with the same speed (10,11). Instead, different regions can be spliced rapidly or slowly (12). A study of pig liver cells showed that for the *pCLEC4G* gene the first intron is spliced simultaneously with several more distal introns, while the second intron is spliced last (13).

Non-sequential intron removal can have unexpected consequences when mutations disrupt splice sites (14). The splicing of the *COL1A1* gene region between exons 5 and 10 can follow two different routes. Removing introns 5, 6 and 9 is always rapid, while the excision of intron 8 can be before or after intron 7 (15). Additionally, point mutations in splice signals were shown to cause skipping of exon 8, inclusion of intron 8 or inclusion of both introns 7 and 8. It is not clear yet which factors control the order of splicing. Theoretically, it might be influenced by the presence of intronic and exonic splice suppressors or enhancers, the ‘strength’ of the donor and acceptor splice sites and/or the size of the intron (16,17).

Recursive splicing is another recently acknowledged feature of the splicing process. The principle of recursive splicing is that an intron might not be spliced in one piece. Instead, the spliceosome can recognize an intra-intronic sequence opposed to the canonical exon–intron border as a splice site, hereby removing the adjacent exon. The rest of such a partly spliced intron will be removed later, at once or again in multiple pieces following the strategy described above. Another type of multi-step intron removal (18) involves the usage of non-canonical donor and non-canonical acceptor splice sites. In this case an inner piece of intron is spliced first and a semi-stable lariat loop structure is formed and will be degraded later (19).

Alternative splicing cannot be fully understood when only mature mRNA is analyzed. When pre-mRNA molecules from different stages of splicing (pre-, intermediate- and post-splicing forms) are captured, the process of splicing can be studied in more detail and previously unknown splicing events can be identified. This type of analysis has been very challenging until recently. However, the development of high-throughput NGS, which involves highly parallelized sequencing of DNA or RNA, enabled the whole transcriptome analysis at high resolution (20–22). In contrast to microarray analysis, RNA-Seq is a non-targeted approach, allowing the discovery of novel splicing events. Nevertheless, the analysis of RNA-Seq data is still a challenge, since NGS experiments generally produce millions of relatively short fragments (reads), even after paired-end sequencing came into play (23). The distance between two sequenced ends of a read pair (‘PE distance’) can be calculated and further taken into account during alignment. Standard mRNA analysis of (paired-end) RNA-Seq data includes mapping reads to the reference sequence, assembling the transcriptome and determining the transcripts (transcript deconvolution) often followed by counting transcript levels (transcript quantification).

While mRNA analysis programs and pipelines are suitable for finding novel exons and exon–exon junctions, they do not consider the presence of pre-mRNA. Instead they analyze the end result of the splicing process, using mainly reads mapped to exons. For this reason these tools are not able to properly deal with mixtures of pre- and mature mRNA, such as found in nuclear RNA extracts.

In this paper we are showing that splicing mechanisms can be analyzed using RNA-Seq data in more detail than previously achieved. We present *SplicePie*—a pipeline which contains a new, dedicated method to analyze the order of splicing and pinpoint putative introns undergoing recursive splicing. Applying this method we show that non-sequentially spliced introns can be identified even in a relatively fast spliced gene. We also identify non-sequentially spliced introns in a gene that have never been reported to undergo such splicing scenarios.

## MATERIALS AND METHODS

### Captured dataset

Fused myotubes from a healthy human muscle cell line were harvested and the nuclei were separated from the cytoplasm using a sucrose containing lysis buffer, Dounce homogenizer and ultracentrifugation, respectively. Nuclear and total RNA were isolated from the nucleus using the Nucleospin RNAII column from BioKe Kit. DNAse treatment (RNAse-Free DNAse set by Qiagen) was performed to avoid DNA contamination. Three micrograms of each sample was reverse transcribed into complementary DNA (cDNA) (SuperScript II reverse transcriptase by Invitrogen), fragmented to the range of 100–600 bp by sonicating these samples with two cycles of 1 min (Covaris S220, Massachusetts, USA) and purified (QIAquick PCR purification kit by Qiagen).

Capture of target sequences was done following the SureSelect XT Target Enrichment System for the Illumina Paired-End Sequencing Library (Agilent Technologies). Illumina adapters were ligated to the fragmented sequences after end repair and A-base tailing (blunting). Further purification steps (performed with the Agencourt AMPure XP beads in 1:1 ratio) eliminated unbound adapters and short fragments (shorter than 100 bp).

The library of probes to capture exons, introns and flanking regions of the target genes (*FXR1, CKLF* and *ACTB*) was designed with the Agilent Technologies eArray software (http://earray.chem.agilent.com), avoiding areas masked by repeat masker and using partially overlapping probes. The 120 bp length probes were biotinylated and four replicates of each probe were designed to reach the required number of baits per library.

The designed library (Gazzoli, I., Pulyakhina, I., Verwey, N. *et al*. unpublished data) was hybridized with the fragmented cDNA from nuclear RNA and total RNA for 24 h, followed by a washing step and pull down of the biotinylated cDNA probes using streptavidin-coated magnetic beads. Eluted samples were amplified to allow for a multiplexed Illumina run. The samples were quantified with the Agilent 2100 Bioanalyzer and Agilent HS DNA Chip Kit (Agilent Technologies, USA). The samples were diluted to a concentration of 7 pM and loaded onto an eight-channel flowcell and sequenced with the Illumina HiSeq 2000 (Illumina, USA). After sequencing, *fastq* files containing paired-end reads (read length of 100 bp) and the base quality information were generated with CASAVA version 1.1 and used for further analysis.

### ENCODE dataset

An RNA-Seq dataset representing a subset of the long RNA-Seq sequencing from ENCODE/Cold Spring Harbor Lab was obtained from the CSHL Long RNA-Seq downloadable files archive (Long RNA-Seq archive from ENCODE/Cold Spring Harbor Lab repository (http://genome.ucsc.edu/cgi-bin/hgFileUi?db=hg19&g=wgEncodeCshlLongRnaSeq), file identifiers are: wgEncodeCshlLongRnaSeqGm12878NucleolusTotal, wgEncodeCshlLongRnaSeqK562ChromatinTotalRep3, wgEncodeCshlLongRnaSeqK562ChromatinTotalRep4.). This dataset (‘ENCODE dataset’) contains RNA-Seq data from human immortalized myelogenous leukemia cell line, two samples of the chromatin-associated RNA and one sample of total nucleus RNA. These samples were sequenced by the Illumina Genome Analyzer II paired-end sequencing technique with read lengths of 75 and 100 bp.

### General information

Alignment was performed with GSNAP version 2012-07-12 using a probabilistic mapping approach (one alignment per read was randomly chosen in case of multiple mappings). Format conversions were done with Samtools version 0.1.18 (24) and in-house scripts. Statistical manipulations and calculations were performed using R version 2.15.1. The Ensembl (25) gene annotation was used for all post-alignment analyses. Only publicly available software was used for the analysis.

### Classification

After alignment, reads were classified according to their splicing stage. The first classification step determines the type of region that the reads are mapped to: exon, intron, exon–exon junction or exon–intron boundary (Table 1).

**Table 1.** Labels for reads based on the mapping location according to transcript annotation (*j* is bigger than *i*)

Read start	Read end	Label
intron	intron	‘int’ (intronic)
exon_*i*_	exon_*i*_	‘ex’ (exonic)
exon_*i*_	exon_*j*_	‘ex–ex’ (exon–exon junction)
exon	intron	‘ex–int’ (exon–intron junction)

**Figure 1.**
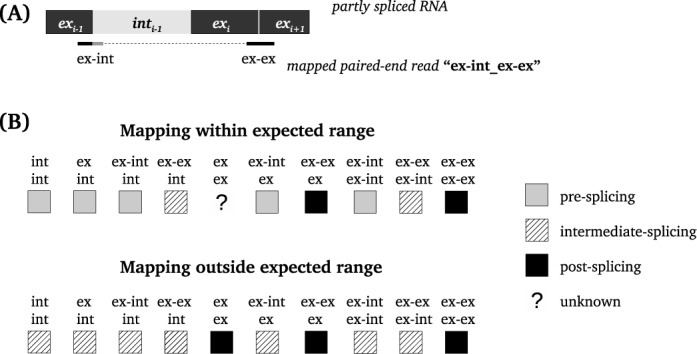
Classification of the paired-end reads. (**A**) ‘Mapping distance’ reflects the inner distance between two read ends according to the genomic coordinates after alignment. (**B**) Classification scheme is built on read labels (‘ex’ stands for exon, ‘int’ for for intron, ‘ex–int’ for intron–exon boundary, ‘ex–ex’ for exon–exon junction) and mapping distance (within/outside expected mapping distance). Reads belonging to pre-, intermediate-, post-splicing and unknown categories are marked with gray, black, striped boxes and a question mark. Example: if one end of the read pair maps to the exon–intron boundary and the other one maps to the exon–exon junction, this read pair will be classified as ‘intermediate’.

Each end of a read pair is given a label (Figure 1) and a ‘mapping distance’. Mapping distance can be calculated for mapped read pairs by measuring the inner distance between the two aligned ends of a read pair.

Read pairs are classified into three categories: pre-, intermediate- and post-splicing. The classification is based on the mapping coordinate information (outside or within the expected distance of around 500 bp) and the labels. The two ends of a read pair are defined as pre-, intermediate- or post-splicing reads. The pre-splicing category contains reads that are partially or fully mapped to intronic regions. The intermediate-splicing category contains read pairs that have both pre-mRNA and mature mRNA characteristics (Figure 1), e.g. one read end maps to an intron, while the other read end maps to an exon–exon junction or to a distant exon. In addition, this category contains reads where two ends are mapped to the same intron or to different introns outside the expected mapping distance range. The post-splicing category contains read pairs indicating that splicing has already occurred in that specific region, e.g. read ends that are mapped to different exons or span an exon–exon junction. When both paired reads map to the same exon, this pair cannot be assigned to any category, as exons are present in all stages of mRNA splicing. These cases are therefore classified separately in the ‘unknown’ category.

Apart from classifying reads into three main categories, we divide them further into more specific subgroups. Read pairs classified as intermediate-splicing reads are used for the analysis of sequentiality if one end is mapped to the exon–exon junction and the second end is mapped to the adjacent upstream or downstream intron. Read pairs having ends that are split across anything but an annotated exon–exon junction are used to identify recursive splicing (ends of a pair are treated separately and the connection between the ends is not considered). Ends of read pairs from the pre-splicing category mapped to the exon–intron boundary and read pairs from the post-splicing category (mapped to the exon–exon junctions) are used to calculate the Splice Site Index (SSI).

### Detection of non-sequential splicing

Non-sequential splicing is approached from two angles: coverage-based and read-based approaches.

For the coverage-based approach the difference between the median coverage of intron_*i* + 1_ and intron_*i*_ is reported (Figure 3). We select negative differences and define the cutoff of significance as the first quartile *Q1* (25% percentile). If the difference is bellow *Q1*, this is an indication that intron_*i* + 1_ is non-sequentially spliced before intron_*i*_. This is calculated for every input sample. If a certain difference is consistent across a number of samples, the corresponding pair of introns is reported.

The read-based approach addresses read pairs that indicate whether two introns are spliced sequentially or non-sequentially (Supplementary Figure S1).

For this we defined the *splice-ratio*, a value that reflects the fraction of reads supporting sequential splicing:

\begin{equation*} {\rm splice}\mbox{-}{\rm ratio} = \frac{{\it {\rm seq}}}{{\it {\rm seq}} + {\it {\rm non-seq}}} \end{equation*}

Here non-seq is the number of reads supporting non-sequential splicing and seq is the number of reads supporting sequential splicing of two adjacent introns. When introns are spliced sequentially, the splice-ratio will be close to 1. However, when a downstream intron is spliced before an upstream intron, the splice-ratio will be close to 0.

We improve the accuracy of the predictions by assessing how often independent read pairs support the same pair of introns being spliced sequentially. Single events might indicate false positives due to mapping artifacts (Figure 3).

### Detecting recursive splicing

We hypothesize that if an intron undergoes recursive splicing, split reads could correctly map across intermediate-splicing products. Recurring observations of these specific products confirm the existence of partially spliced introns. To extract such reads, we first identify potential hotspots for recursive splicing (Supplementary Figure S2). For each position we calculate how many times a read has been split over it (in other words, we calculated the ‘inverted coverage’—coverage of gaps) and then calculate the derivative of this inverted coverage. The derivative indicates where the inverted coverage changes relatively to the previous positions, implying how many reads share a breakpoint at that specific location. Positive values represent a split's start, while negative values indicate a split's end. All other positions will have a derivative value of zero. Each peak is reported in a *wiggle* file. Positions which are start- and endpoints for splits at the same time are excluded from this analysis. Reads spanning exon–exon junctions are also removed, as they indicate annotated exon–exon junctions and do not contribute to the investigation of recursive splicing.

In order to reduce the amount of noise and false positives, we create *wiggle* files for all input samples and evaluate the overlap in the requested number of samples. If the position has positive coverage in a number of samples, the sum of the coverage from all files will be reported. This results in a single file with the most robust positions. Reads covering the positions from the final list are extracted from the initial bam file(s) and analyzed to validate the prediction of recursive splicing hot spots from the *wiggle* file and get the connections between the peaks (which are lost in the *wiggle* file).

Additionally, a text file containing a matrix with all discovered junctions and the number of reads supporting each junction is created per gene (Figure 4).

### Calculating SSI and processing the coverage

We developed the SSI, a value used to detect splicing events, which is calculated in the following way:

\begin{equation*} {\rm SSI} = \frac{{{\rm ex-ex}}}{{{\rm ex-ex}} + {{\rm ex-int}}} \end{equation*}

Here SSI is the splicing index value, ex–ex is the number of reads spanning exon–exon junctions and ex–int is the number of reads spanning exon–intron junctions.

A similar function, called completed Splicing Index (coSI), has been recently developed (26). In contrast to coSI, SSI is calculated separately for 5′ and 3′ splice sites of each intron, allowing for the assessment of (i) the relative abundance of each intron and (ii) whether both ends are spliced simultaneously. SSI, in combination with the median coverage of introns and exons, gives a more complete picture of the alternative splicing events. SSI is calculated as a ratio of different types of reads and does not consider the difference between the absolute values of coverage.

### Experimental validation of non-sequential and recursive splicing

cDNA from three different healthy muscle cell lines (also used in the *in silico* analysis) was generated using the reverse transcriptase, 1 μg of pre-mRNA and random primers (following the standard protocol suggested by SuperScript®III RT by Invitrogen).

For the validation of non-sequential splicing, forward primers were designed against exon 10 or intron 11. Reverse primers were designed against exon 11 or the junction of exon 11 and exon 12. All combinations of primers were used to detect all splicing intermediates that can be formed in this area of the gene. Each sample was analyzed with three technical replicates and normalized against the HPRT gene. Quantitative polymerase chain reaction (QPCR) was performed on the LightCycler 480 (Roche Diagnostics Ltd.) using SYBR Green mix. QPCR results were analyzed using LightCycler 480 and LinRegPCR software (27). Independent amplification, with primers in intron 10 and exon 12, was performed for further Sanger sequencing analysis.

For the validation of recursive splicing events, we amplified the same synthesized cDNA template using a pair of primers located upstream of the predicted donor and downstream of the predicted acceptor splice sites. For the event with one non-annotated splice site the forward primer was located in exon 16 and the reverse primer was located in intron 16. For the event with both non-annotated splice sites both primers were located in intron 16. Polymerase chain reactions (PCRs) were performed with 35 cycles using Phusion®High-Fidelity PCR 2X Master Mix with HF Buffer (New England BioLabs Inc).

The amplification products were then separated on a 1.5% agarose gel after an RT-PCR reaction. The dissected bands were extracted and eluted (MinElute gel extraction kit, Qiagen) and sequenced with Sanger sequencing.

## RESULTS

### Pipeline overview

**Figure 2.**
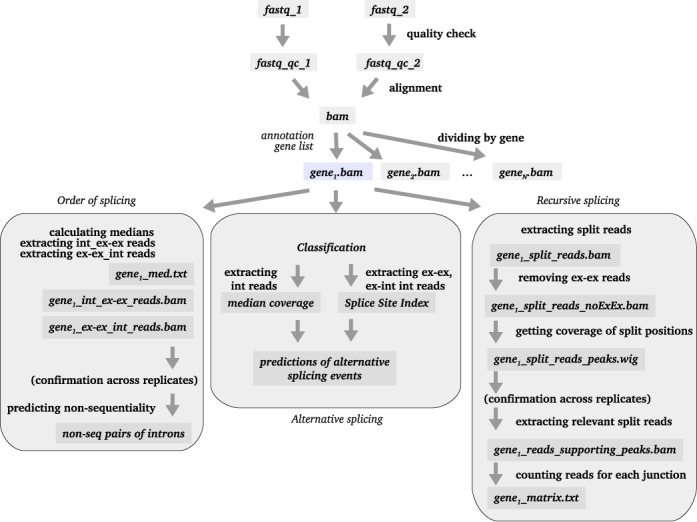
Layout of *SplicePie*. Light-gray boxes indicate the files required/produced during the mainstream analysis. Labels in ‘bold’ next to the arrows indicate the steps of analysis. Labels in ‘italic’ next to the arrows indicate the additional input files. Label *int_ex–ex* indicates that the file contains read pairs with one end mapped to the intron and the other end mapped to the exon–exon junction (and vice versa for *ex–ex_int*).

*SplicePie* starts with a standard quality check procedure, removes low quality reads and performs split read alignment to the reference genome. The gene of interest is then extracted from the alignment file and is used to calculate the SSI and classify reads based on their stage of splicing (pre-, intermediate- and post-splicing, see Section “Classification" in “Materials and Methods" 3.4 and Table 1 for details). Reads from specific categories are used to predict and pinpoint putative non-sequentially or recursively splice introns. An overview of *SplicePie* is shown in Figure 2.

Alignment to the reference genome (hg19, GRCh37) is performed using GSNAP (28). This is a fast, Single Nucleotide Polymorphism (SNP)-tolerant tool that works with paired-end RNA-Seq data and can split each read end into multiple fragments, thereby coping effectively with a gene's intron/exon structure. We have chosen this tool over Tophat (29,30), PASSion (31), HMMSplicer (32) and MapSplice (16) because, unlike these tools, GSNAP does not give priority to split alignments. This is crucial for pre-mRNA data, as such data is expected to contain reads across exon–intron boundaries. Unlike other tools, it uses information about canonical and non-canonical splice site signals when splitting the reads, which is important for the identification of novel exons. It is also able to split each read of the pair into as many fragments as necessary (in case of multiple adjacent small exons). GSNAP provides the results in the commonly used *sam* format. *SplicePie* generates *bam* and *wiggle* files from these *sam* files. Our analysis approach is mostly suitable for very detailed analysis, therefore it is recommended to analyze one gene of interest at a time. However, running *SplicePie* for multiple genes is also supported and all gene annotations provided by the user in a standard *GTF* format will be used to build a list of the genes of interest. *SplicePie* performs the classification of reads as pre-, intermediate- or post-splicing according to their mapping position (see Section “Classification" in “Materials and Methods" for details). After classification all reads spanning a specific exon–exon junction or an exon–intron boundary are used for the SSI calculation. SSIs will then be calculated for each splice site (see Section “Calculating Splice Site Index and processing the coverage" in “Materials and Methods" for details). The output is provided as a *text* file containing SSIs for both 5′ (SSI^5^) and 3′ (SSI^3^) ends per intron. SSI value is calculated using the reads spanning exon–exon junctions and exon–intron boundaries. This value is similar to the coSI (26), which reflects the amount of RNA molecules containing the exon with spliced adjacent introns. However, SSI is calculated separately per splice site and therefore reflects the difference between 5′ and 3′, thereby highlighting alternative splicing and/or incomplete splicing (while coSI reflects the completion of the splicing of a particular intron).

**Figure 3.**
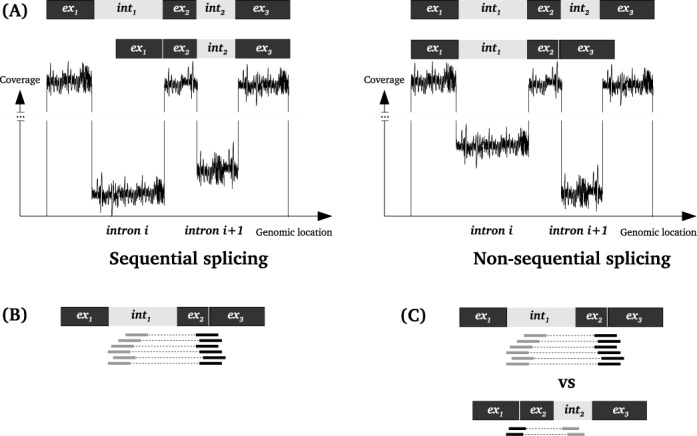
Principles of the two approaches to investigate non-sequential splicing. (**A**) Coverage-based approach with the underlying assumption that the longer the intron is present in the sample, the higher the coverage will be. In case of non-sequential splicing the coverage of the downstream intron_*i* + 1_ is likely to be lower than the coverage of the upstream intron_*i*_. Median of the coverage of each intron is used in this approach. (**B**) The underlying assumptions for the read-based approach to detect non-sequential splicing: evidence for non-sequential splicing is obtained from read pairs with one end mapped to the upstream intron_*i*_ and the other end mapped to the junction over the downstream intron_*i* + 1_ (exon_*i* + 1_-exon_*i* + 2_ junction). (**C**) Method to select the introns with non-sequential splicing. The read pairs supporting the splicing intermediate where intron_*i*_ is spliced before intron_*i* + 1_ should be less abundant than the read pairs for the intermediate product where intron_*i* + 1_ is spliced before intron_*i*_.

A parallel branch of *SplicePie* is analyzing the order of splicing and predicts which introns are spliced sequentially and which introns are spliced non-sequentially. Median coverage of each intron is calculated and when the difference between the downstream intron_*i* + 1_ and the upstream intron_*i*_ is low, such pair of introns is potentially non-sequentialy spliced. At the moment we define this value as ‘low’ when it is below *Q1*, where *Q1*, or lower quartile, is defined as the middle number between the smallest number and the median of the dataset (Figure 3A).

In addition to the coverage information we consider reads supporting or contradicting non-sequential splicing (Figure 3B and C). In case when one end of a paired-end read is mapped to the upstream intron_*i*_ and the other end of the same pair is mapped across the downstream splice junction over intron_*i* + 1_, such pair is consistent with non-sequential splicing. When one end is mapped across the splice site of the upstream intron_*i*_ and the other end is mapped to the downstream intron_*i* + 1_, such paired-end read is consistent with sequential splicing. We calculate *splice-ratio*, the fraction of reads supporting sequential splicing, and the lower this ratio is, the higher is the probability that introns are non-sequentially spliced. Therefore, low splice-ratio in combination with big difference in coverage (see above) pinpoints introns that are potential targets of non-sequential splicing.

**Figure 4.**
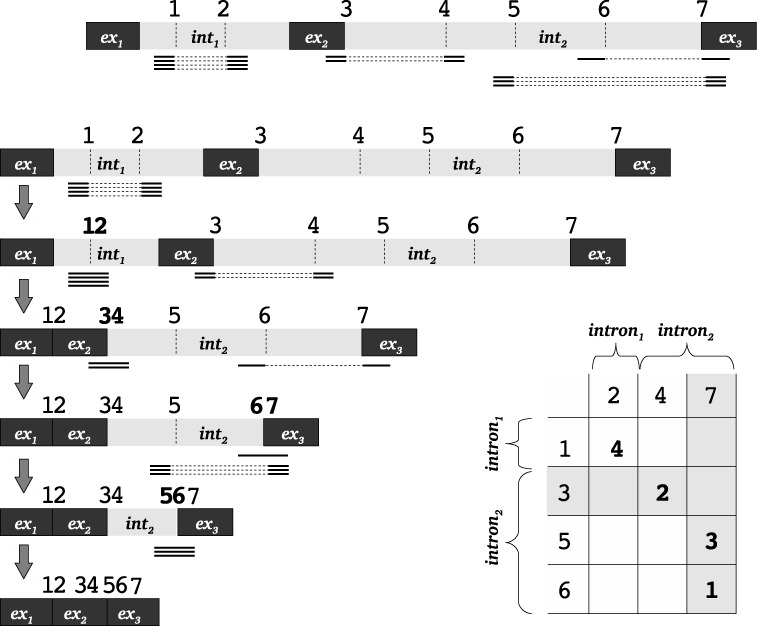
Graphical representation of the analysis of recursive splicing. Black boxes represent exons and gray boxes represent introns, dashed lines across the introns and exon–intron borders represent positions of splits in reads. Numbers ‘1’, ‘2’, etc., on top of the gene schema represent the positions of the gene in genomic coordinates. Thick lines connected with dashed lines represent split reads (where the dashed part of the line represents the area across which the read is split). Step-by-step analysis of all split reads is shown and splicing intermediates corresponding to each group of split reads are shown on the schema in left part of the figure. The matrix in the right bottom corner contains donor splice sites (top row) and acceptor splice sites (left column). Each cell in the matrix represents the number of reads supporting such junction. Numbers in the gray cells of the matrix represent reads with one new non-canonical splice site. Numbers in white cells of the matrix represent reads with two new non-canonical splice sites (reads split within an intron).

Another branch of *SplicePie* processes the *wiggle* files and pinpoints introns potentially undergoing recursive splicing. The *wiggle* files contain only the coverage of splice sites. We take all combinations of donor and acceptor splice sites and calculate the number of reads supporting each pair. This creates a summary matrix containing all potential recursive splicing events and their frequency in each sample (Figure 4).

### Alternative splicing events in captured dataset

**Figure 5.**
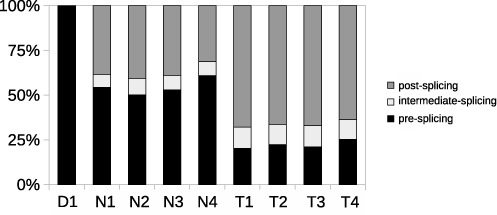
Classification of reads from the capture dataset mapped to *FXR1*. For sample identifiers see Table 2. The figure displays the percentage of reads mapped to *FXR1* classified into pre-, intermediate- and post-splicing fractions for pre-mRNA and total RNA samples in the captured dataset.

To study pre-mRNA processing, four nuclear RNA, four total RNA and one DNA sample (Table 2) of specific genes were sequenced (see Section “Captured dataset" in “Materials and Methods" for details). The analysis of the target gene—*FXR1*—will be discussed in this section. From the targeted genes, we have studied the pre-mRNA splicing of *FXR1* (70 306 nt long) in detail because it is known to be alternatively spliced, has an intermediate number of exons (18), introns with variable length between 86 and 18 338 nt and a decent intronic coverage (on average above 1500 for nuclear RNA samples) in the cell lines analyzed (Table 2). Please note that the numbers of reads for the categories do not always add up to the total number of reads mapped to the target, since we do not show the number of reads defined as ‘unclassified’ (see Section ‘Materials and Methods’). The classification of reads into three categories supporting pre-, intermediate- and post-splicing events is used to estimate the pre-mRNA content of samples (see Section “Captured dataset" in “Materials and Methods" for details). Compared to the total RNA samples, all nuclear RNA samples contain a larger fraction of reads coming from the pre-splicing stage (Figure 5). This is expected, as total RNA is isolated from the whole cell and consists mostly of mature mRNA, while nuclear RNA contains (partly) spliced RNA. The post-splicing category contains a large fraction of reads even in nuclear RNA samples, which might be a consequence of the fast splicing of *FXR1*, which makes it hard to capture the nuclear pre-mRNA of this gene. Reads from the DNA sample were mainly classified as ‘pre-splicing’, which is expected, since DNA is not supposed to contain any exon–exon junctions.

**Table 2.** Characteristics of samples and summary of results for reads mapped to *FXR1*

Sample name	Description	Reads mapped on target	Norm. pre-splicing	Norm. interm.-splicing	Norm. post-splicing	Large interm.-splicing	Large post-splicing
D1	DNA	306103	286501	148	29	209	6
N1	**n**uclear RNA1	317827	139084	11834	64750	6879	33688
N2	**n**uclear RNA2	586827	224231	28520	134229	12336	47954
N3	**n**uclear RNA3	431321	184823	15845	91217	12456	44680
N4	**n**uclear RNA4	1394314	707109	51856	222918	39003	141707
T1	**t**otal RNA1	242979	41232	11347	81826	9456	41048
T2	**t**otal RNA2	181383	28002	9234	62588	7222	31376
T3	**t**otal RNA3	261004	42412	10932	67126	13111	68536
T4	**t**otal RNA4	317123	60915	12528	81132	13901	72375
C1	**c**hromatin RNA1	21632	19445	169	489	77	209
C2	**c**hromatin RNA3	8600	7251	62	189	39	91
U1	n**u**cleolic RNA	20283	16566	173	152	123	158

For each sample the reads were classified as pre-, intermediate- or post-splicing based on the distance between paired ends (<650 being normal and ≥650 being larger than anticipated). Samples D1-T4 are from the captured dataset, samples C1-U1 from the ENCODE dataset.

#### SSI and medians of coverage

In order to investigate alternative splicing events we use a combination of SSI values and medians of intronic and exonic coverage.

As the coverage may be influenced by the probe hybridization efficiency, we evaluated the uniformity of the coverage in the DNA sample D1 (Supplementary Figure S4). The only dips in coverage take place in the Repeat Masker regions, which were excluded from probe design. To confirm the limited influence of probe hybridization on coverage, we calculated the standard deviation of the median coverage per intron, which was only 11.52% of the average.

**Figure 6.**
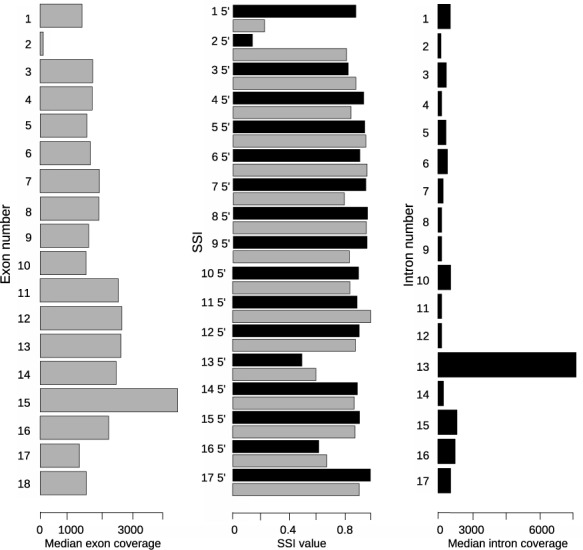
SSI and medians of coverage of exons and introns in *FXR1*. Gray bars in the left panel represent the coverage of exons (exon 1 on top). Black bars in the middle panel represent SSI^5^ values the introns and gray bars on the middle plot represent SSI^3^ values of the introns (intron 1 on top). Black bars in the right plot represent the coverage of introns (intron 1 on top). Data shown for nuclear RNA sample N2.

Median coverage has been calculated per intron, while SSI is calculated per 5′ and 3′ end of each intron separately. SSI can indicate various alternative splicing events, e.g. (partial) intron retention and exon skipping (data shown for sample N2, Figure 6). Decreases in the SSI^5^ of intron 2 and in the SSI^3^ of intron 1 are indicative of the skipping of exon 2 in a subset of transcripts. This is confirmed by the relatively low coverage of exon 2. The coverage of intron 13 is significantly higher than the average intronic coverage, which can indicate intron retention in both pre- and mature mRNA. This is also supported by low SSI^5^ and SSI^3^ values of intron 13, which means that the number of reads mapped to the boundary of exon 13 and intron 13 and the boundary of intron 13 and exon 14 are over-represented. The retention of intron 13 and skipping of exon 2 were experimentally confirmed for sample N2 (Supplementary Figure S4). Thus, low SSI^5^ and SSI^3^ on the same intron are the indication of an intron retention, whereas low SSI^3^ of an intron in combination with low SSI^5^ of the next intron is an indicator of exon skipping.

We also developed a module to rank introns based on their probability to be retained. For retained introns, both SSI^5^ and SSI^3^ values should be low. To estimate that, we calculate the magnitude of SSIs (how big the difference between SSI^5^ and SSI^3^ values is) and the likelihood of each magnitude (Supplementary Figure S5). The introns with the highest magnitude and a low *P*-value are the main candidates for retention (Supplementary Table S1).

### Non-sequential splicing in captured dataset

In the captured dataset, we searched for non-sequentially spliced introns in *FXR1*. Multiple candidate pairs of introns with a difference in median coverage below *Q3* were found: introns 1 and 2, introns 3 and 4, introns 10 and 11, introns 16 and 17. (Here Q3 stands for the third quartile—the middle value between the median and the highest value of the data.) To confirm this, we calculated the ratio of read pairs in support over those not in support of non-sequential splicing. If the splice-ratio is close to 1, the splicing is most likely sequential, the lower the ratio is, the more non-sequential splicing occurs. We observed a very strong correlation between both methods, supporting the idea that these methods are suitable to identify non-sequentiality spliced introns (Pearson *R*^2^ ≤ 0.86 and Spearman *R*^2^ ≤ 0.85) (Supplementary Figure S6).

**Figure 7.**
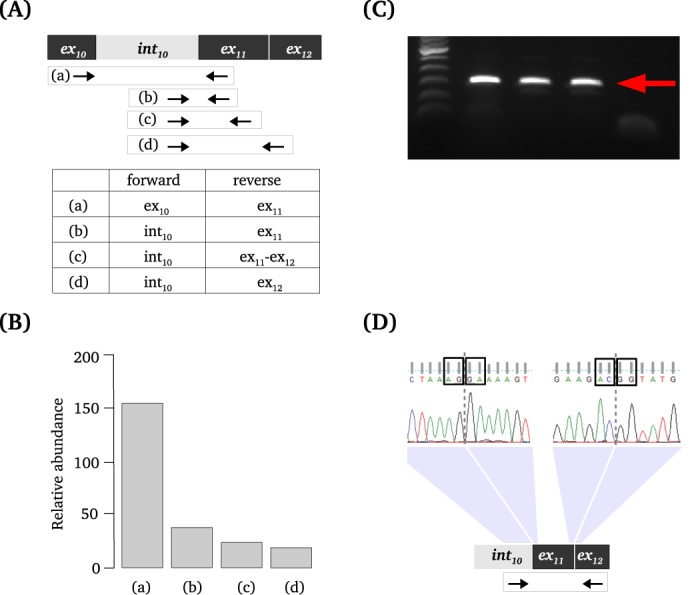
Experimental validation of predicted non-sequential splicing of introns 10 and 11 in *FXR1*. (**A**) The design of the primers for the validation of non-sequential splicing of intron 10. (**B**) The results of quantitative real-time PCR showing the relative abundance (on the Y axis) of splicing intermediates with primer combinations described in (A) (on the X axis). Since three tested cell lines showed similar reproducible results, the figure shows the average. (**C**) Results of PCR showing the presence of the fragment of anticipated size. Lane 1 contains the marker, lanes 2, 3 and 4 represent three cell lines, lane 5 shows the negative PCR control. (**D**) The results of Sanger sequencing of the band shown on (C). The top panel shows the output of Sanger sequencing, black box around ‘AG’ depicts the acceptor splice site, the boxes around ‘GA’, ‘AC’ and ‘GG’ depict ends of the exons. The bottom panel shows the design of the primers for the sequenced amplicon.

We used DNA as a negative control, since DNA does not undergo splicing and the coverage of the introns should not differ significantly. If introns have significantly different coverage on DNA level, this may be due to sequencability, mappability bias or other technical artifacts (not a biological reason). Two pairs of introns predicted to undergo non-sequential splicing in RNA samples (introns 3 and 4, introns 10 and 11) survived this extra control step. They were not classified as non-sequentially spliced in DNA. Introns 10 and 11 have been selected for further the experimental validation and showed to be non-sequentially spliced (Figure 7).

### Recursive splicing in captured dataset

We searched for the evidence of multi-step splicing in our captured dataset (as described in Section “Detecting recursive splicing" in “Materials and Methods"). *Wiggle* files containing coverage at positions where the reads were split have been created for four pre-mRNA samples (reads spanning exon–exon junctions were excluded). We considered positions present in all samples with the same sign for further analysis, because they were deemed most reliable.

We assessed the distribution of peak coverage and tried to identify the minimal coverage for the peak to be included in the final list. We first investigated the highest peaks and found out that the coverage of the regions between the peaks is higher compared to the rest of the introns. These observations let us hypothesize that such intronic regions with high coverage and split reads mapped to them and to the adjacent exons might be novel exons. We were able to experimentally validate one of potential novel exons (Supplementary Figure S7). Our findings thus suggest that the method developed for the detection of recursive splicing events is also suitable for finding novel exons.

After excluding the peaks with the highest coverage we found eight events to be recurrent and consistent across all RNA samples (Supplementary Table S2). These events were not present in the DNA sample D1, which had only 61 split reads (against hundreds of split reads in RNA samples), none of the split positions was supported by more than one read. The vast majority of split reads in DNA were mapped with a large number of mismatches (unlike split reads in RNA samples).

The most abundant events of recursive splicing occurred on the 5′ end of the intron (when the donor splice site is canonical and the acceptor splice site is situated within the downstream intron, Supplementary Figure S8A). The second biggest class (three cases, or around 40%) were the cases of recursive splicing occurring on the 3′ end of the intron. We also found a pair of events with shared canonical acceptor splice site (the intron 10-exon 11 boundary), which suggests the possibility of multi-step recursive splicing in this area (Supplementary Figure S8B). Furthermore, we were also able to identify one recurrent event of inner recursive splicing with two non-canonical splice sites (Supplementary Figure S8C).

The strength of the newly identified donor or acceptor splice sites associated with potential recursive splicing events was assessed with Human Splicing Finder (http://www.umd.be/HSF3/HSF.html). All sites were identified as highly probable putative splice sites.

To obtain further evidence that the recursive splicing events identified by *SplicePie* are genuine, we evaluated the sequence motifs flanking the splits. We selected events present in two, three, four or five out of five samples and extracted the splice sites and two nucleotides upstream of the donor and downstream of the acceptor sites. We calculated the percentage of canonical and non-canonical donor and acceptor splice sites in the events with non-annotated donor or acceptor splice site. In this analysis we omit potential recursive splicing events with both non-annotated splice sites, as they are more likely to be novel exons. Such events require further experimental validation, and only experimentally showing there presence in pre- and possibly mature mRNA will distinguish between recursive splicing and novel exons.

Our results (Supplementary Table S3) show strong enrichment for canonical donor (GT) and acceptor (AG) splice sites for events with one non-annotated splice site present in five samples. The enrichment becomes weaker for the events detected in only a subset of the samples, especially for the events with a non-annotated acceptor site. This indicates that the recurrent events with one non-annotated splice site are the most robust recursive splicing events. Please note that the aligner we use does not have any preferences for splice motifs when splitting reads.

Apart from the *in silico* validation, we also used RT-PCR followed by Sanger sequencing to validate potential recursive splicing events. We designed primers flanking two events found in all five samples and analyzed nuclear and cytoplasmic RNA isolated from two of the muscle cell lines. For the first event (chr3:180,689,975-180,692,201, Supplementary Table S2), detected a product of the expected length and sequence. However, it was found in both nuclear and cytoplasmic RNA, suggesting that the detected event represents a novel exon and not an intermediate-splicing product. The second set of primers captured another recursive splicing event that was not present in the RNA-Seq data.

### Performance on non-targeted dataset

To demonstrate the performance of *SplicePie* on regular, non-targeted RNA-Seq data, three samples from the Gencode project (the ENCODE dataset) containing RNA from different nuclear compartments (chromatin-associated and nucleolus RNA) were analyzed following the same procedure as the captured dataset. Around 95% of reads mapping to the *FXR1* gene were classified as pre-splicing for chromatin and nucleolus RNA in contrast with 20 and 60% for the nuclear and total RNA samples, respectively. This is in line with the presence of pre-mRNA in chromatin and nucleolus, while mature mRNA is prevailing in the nucleoplasm.

The values of SSI and medians of exonic and intronic coverage for the non-targeted RNA dataset (Supplementary Figure S9) coincide with corresponding values calculated for the captured dataset. Low SSI^3^ for intron 1 and low SSI^5^ for intron 2 indicate the skipping of exon 2 and high coverage of intron 13 together with its low SSI^5^ and SSI^3^ are indicative of intron 13 retention. However, the pattern of the medians is less consistent for both exonic and intronic coverage. The C1 sample contains chromatin-associated RNA, for which splicing is not known to be in action, the difference between the exonic and intronic coverage is small, therefore the coverage values for chromatin-associated RNA are less informative than those for nucleoplasmic RNA.

We were able to confirm the previous findings (of intron 10 and intron 11 being non-sequentially spliced) using the approach based on median intron coverage (see Section “Detecting non-sequential splicing" in “Materials and Methods" for details). Other predictions were mostly confirmed in at least two out of three analyzed samples with the coverage-based approach (data not shown). However, the number of reads needed to calculate the splice-ratio was not high enough, so the majority of the ratios equaled zero.

The non-targeted RNA dataset was also analyzed in order to find potential recursive splicing events. Five peaks were present in all three samples. Two of these peaks were found in the list of donor/acceptor splice sites identified for the captured dataset. The number of splice sites identified per sample was ∼100, while the number of peaks for each sample of the captured dataset was over 450.

In order to demonstrate the performance of *SplicePie* on the whole-transcriptome dataset, we selected *TIA1* for the analysis of non-sequential and recursive splicing.

Based on the difference in median intronic coverage (which was at least three times higher than the upper quartile (75%) in all three samples) and high splice-ratio (average of 0.7 in the three samples), we could predict two introns of *TIA1* to be non-sequentially spliced. Neither intron 2 nor intron 3 is overlapping with any genomic elements that might influence the coverage, such as pseudogenes or non-coding RNAs.

According to both considerable difference in coverage and high splice-ratio, intron 3 is predicted to be spliced before intron 2 (Supplementary Figure S10). Even more cases of potential non-sequential splicing were found in two out of three samples, however, to claim that the order of splicing of these introns is non-sequential, experimental follow-up is required.

We also investigated multi-step splicing in *TIA1* and were able to detect a number of potential recursive splicing events. Events present in all three samples C1, C2 and U1 with non-annotated donor, non-annotated acceptor and both non-annotated splice sites were detected (Supplementary Table S4).

Therefore, non-targeted total RNA-Seq data provides sufficient information to analyze recursive splicing for some genes and can be used for the detailed investigation of splicing in action.

## DISCUSSION

Exploring pre-mRNA processing is facilitated by new sequencing technologies reaching higher throughput, hence producing more data. The analysis of both pre- and mature mRNA provides new insights into splicing mechanisms and alternative splicing events. However, current software is focusing on mature mRNA and the identification (and quantification) of transcript variants.

The presented pipeline for the pre-mRNA data analysis, *SplicePie*, offers a number of approaches and solutions to study splicing in more details. The proposed strategy performs well on different sample preparations (sequencing the whole pool of RNAs or capturing a gene with different relative quantities of pre- and mature mRNA). Our method can detect various genuine alternative splicing events like intron retention, exon skipping and novel exons. Furthermore, it is capable to resolve the order of splicing and recursive splicing events.

The methodology of *SplicePie* significantly differs from existing pipelines, such as Cufflinks, Scripture (33) or MISO (34). These tools focus their analysis on the end result of splicing and mainly use reads mapped to the exons or exon–exon junctions. Reads mapped to the introns are either treated as putative exons or not addressed at all (in case of annotation-based pipelines). Therefore these pipelines are not able to analyze mixtures of pre- and mature mRNA (as found in nuclear RNA extracts). This is crucial to understand the details of the splicing mechanism. Our pipeline *SplicePie* is specifically geared toward the analysis of the full splicing process in action. In order to do so, it uses all reads mapped to exons, introns, exon–exon junctions or exon–intron boundaries.

For the analysis of alternative splicing events, the SSI and the medians of exonic or intronic coverage methods implemented in *SplicePie* are mutually reinforcing. In case of captured data enriched with partly spliced nuclear RNA, the difference in exonic and intronic coverage makes the patterns of coverage informative for assessing alternative splicing events. In case of ‘pure’ nuclear RNA with lower abundance of spliced fragments, the difference in exonic versus intronic coverage drops, however, the SSI values become more informative.

The main novelty of the methodology introduced in this paper is the possibility to analyze splicing order and the stepwise nature of splicing. While we are able to judge local splicing order, i.e. one intron is spliced before a neighboring intron, it is not possible to determine the global order of splicing for the entire transcript. This happens due to the co-transcriptional nature of the splicing process and the fact that we capture only one snapshot of the nascent transcript. We show that the local order of splicing for certain introns within *FXR1* is reproducible (in biological replicates) and even consistent across multiple cell lines. Furthermore, this can be confirmed with independent PCR-based technologies.

Although the fact that splicing can be performed in multiple steps has been known for over a decade (35,36), however, it has never been analyzed bioinformatically. We show that analyzing the intermediate category of reads is possible for both potential novel exons and recursive splicing events. However, focusing on a narrow fraction of reads might result in analyzing random events, which is why we suggest to use as many samples as possible. To improve reliability even further it is advised to select events occurring in a significant number of samples. This strategy also helps reducing biases introduced by PCR duplicates, which are not likely to appear at the same position in replicates. The splice motif analysis of potential recursive splicing events provides evidence that these events are genuine, considering the canonical splice motifs and the occurrence of the events across multiple cell lines. However, as RNA-Seq is more sensitive than PCR, not all detected events can be experimentally confirmed at the moment. Recursive splicing events with both non-canonical splice sites should be treated especially carefully, as for these events it is hard to distinguish between recursive splicing and novel exons without the experimental validation in both nuclear and cytoplasmic RNA.

Detecting background noise is a common problem in bioinformatics and statistics, especially when working with large datasets containing a mix of introns and exons. Our approach was shown to perform well on both high (captured dataset) and low (non-targeted ENCODE dataset) coverage data. Moreover, despite the combination of low coverage and noise, alternative splicing events were detected reliably. This indicates that total RNA sequencing can be used for detecting non-sequential splicing events relying mainly on coverage information. *SplicePie* can be run on any dataset, however, the main concern is the average coverage of the introns. Recent total RNA sequencing protocols do not provide enough intronic coverage to perform the analyses with the same power as the analyses on captured data. Another concern is that some genes are not transcribed and expressed highly enough, therefore their coverage will be low unless a library to capture and enrich for these particular genes is designed. Our study shows that total RNA sequencing of specifically nucleus does not generate enough coverage to detect non-sequential and multi-step splicing as efficiently as captured RNA libraries. We would still recommend to do the targeted sequencing of the genes of interest to allow a more in-depth analysis.

Using *SplicePie* on different datasets revealed various not yet annotated splicing events. Our work enhances the value of pre-mRNA sequencing data and pioneers the investigation of the mechanisms of (alternative) splicing.

## SUPPLEMENTARY DATA

Supplementary Data are available at NAR Online.

SUPPLEMENTARY DATA
