# SplicePie: a novel analytical approach for the detection of alternative, non-sequential and recursive splicing

**DOI:** 10.1093/nar/gkv1062

**Published:** 2015-10-07

**Authors:** Irina Pulyakhina, Isabella Gazzoli, Peter A.C.'t Hoen, Nisha Verwey, Johan T. den Dunnen, Annemieke Aartsma-Rus, Jeroen F.J. Laros

*Nucl. Acids Res*. 43 (12): e80. doi: 10.1093/nar/gkv242

Johan den Dunnen's middle initial was omitted. The author's full name should be Johan T. den Dunnen.

The publisher apologises to the authors for this error.

