# Supplementary material for: SplicePie: a novel analytical approach for the detection of alternative, non-sequential and recursive splicing
Source: Nucleic Acids Res. 2015 Mar 23;43(12):e80. doi: 10.1093/nar/gkv242 (PMC4499118; doi:10.1093/nar/gkv242)
Supplement: SUPPLEMENTARY DATA [file supp_43_12_e80__index.html]

SplicePie: a novel analytical approach for the detection of alternative, non-sequential and recursive splicing — SplicePie: a novel analytical approach for the detection of alternative, non-sequential and recursive splicing — SplicePie: a novel analytical approach for the detection of alternative, non-sequential and recursive splicing — SUPPLEMENTARY DATA 

# SplicePie: a novel analytical approach for the detection of alternative, non-sequential and recursive splicing

## SUPPLEMENTARY DATA

**Files in this Data Supplement:**

- SUPPLEMENTARY DATA
