# Supplementary material for: SplicePie: a novel analytical approach for the detection of alternative, non-sequential and recursive splicing
Source: Nucleic Acids Res. 2015 Mar 23;43(12):e80. doi: 10.1093/nar/gkv242 (PMC4499118; doi:10.1093/nar/gkv242)
Supplement: SUPPLEMENTARY DATA [file supp_gkv242_nar-00140-met-g-2015-File006.pdf]

SplicePie: a novel analytical approach for the detection of alternative, non-sequential and recursive splicing

I.Pulyakhina[1,*], I. Gazzoli[1], P.A.C. 't Hoen[1],
N.E. Verwey[1], J.T. den Dunnen[1,2], A. Aartsma-Rus[1], J.F.J. Laros[1,2]

1 Department of Human Genetics, Leiden University Medical Center, Leiden, the Netherlands

2 Leiden Genome Technology Center, Leiden University Medical Center, Leiden, the Netherlands

I.Pulyakhina@lumc.nl

February 20, 2015

Supplementary data

Table S1: Predicting intron retention events based on the magnitude of the SSIs and the p-value. Column "$5'$ ex-int" contains number of reads mapped to the exon-intron boundary on the 5'-end of an intron (used to calculate SSI^5). Column "$3'$ ex-int" contains number of reads mapped to the exon-intron boundary on the 3'-end of an intron (used to calculate SSI^3).

intron	$5'$ ex-int	$3'$ ex-int	ex-ex	magnitude	p-value
1	2463	261	3768	0.03	0
2	679	1465	2100	0.14	6.08e-66
3	1202	1452	6821	0.08	1.32e-06
4	712	1571	10189	0.03	7.54e-74
5	769	1406	9705	0.04	6.09e-43
6	2125	940	17086	0.03	4.19e-104
7	324	1667	5493	0.03	3.13e-217
8	574	409	7064	0.03	1.58e-07
9	1034	1894	12403	0.04	1.46e-57
10	2032	3088	20646	0.05	1.54e-49
11	1840	82	12225	0.01	0
12	880	2660	14853	0.03	1.45e-205
**13**	**7783**	**9550**	**7525**	**0.34**	**4.29e-41**
14	1935	3046	18715	0.05	3.44e-56
15	8137	4099	30855	0.06	4.23e-297
16	4602	3264	11903	0.12	1.36e-51
17	786	2272	14427	0.03	1.15e-165

Table S2: Representation of recursive splicing in the captured dataset. Column "coordinates" contains the coordinates of recursive splicing events in the reference genome, column "splice site" contains which splice site is non-annotated, columns "N1", "N2", "N3" and "N4" contain the number of reads supporting each recursive splicing event in each nuclear RNA sample from the captured dataset. All detected events are located on chromosome 3.

Coordinates	Splice site	N1	N2	N3	N4
180,653,019-180,665,633	acceptor	1	1	4	1
180,674,213-180,675,607	donor	2	2	4	2
180,674,835-180,675,607	donor	5	4	2	1
180,680,878-180,681,592	acceptor	5	1	4	2
180,686,042-180,687,934	acceptor	20	73	42	23
180,688,146-180,688,665	acceptor	8	401	85	29
180,689,975-180,692,201	both	5	9	10	3
180,692,935-180,693,101	donor	13	37	26	18

Table S3: Canonical and non-canonical splice sites in potential recursive splicing events. Column "Non-annotated acceptor" contains the information about potential recursive splicing events with a non-annotated acceptor and column "non-annotated donor" contains information about potential recursive splicing events with a non-annotated donor – total number of such type of events and percentage of events that have a canonical acceptor (AGxx) or donor (xxGT) splice site, respectively.

Number of samples	Non-annotated acceptor		Non-annotated donor	
containing an event	number of events	AGxx	number of events	xxGT
Five out of five	4	75%	3	100%
Four out of five	36	86%	28	100%
Three out of five	53	90%	49	100%
Two out of five	78	85%	95	95%

Table S4: Representation of recursive splicing in *TIA1* detected in the non-targeted dataset. Column "coordinates" contains the coordinates of recursive splicing events in on the reference genome. Column "splice site" indicates which splice site is non-annotated. Columns "C1", "C2" and "U1" contain the number of reads supporting each recursive splicing event in each RNA sample from the non-targeted dataset.

Coordinates	Splice site	C1	C2	U1
70,443,631-70,443,885	donor	15	3	61
70,451,761-70,452,460	donor	16	5	7
70,451,761-70,452,597	donor	1	1	1
70,452,525-70,454,867	acceptor	15	3	17
70,454,954-70,455,476	donor	23	15	16
70,455,594-70,456,191	acceptor	20	20	13
70,457,986-70,460,773	donor	4	3	6
70,460,894-70,463,211	acceptor	1	1	2
70,463,307-70,465,921	donor	5	2	2
70,469,796-70,469,830	both	5	3	2

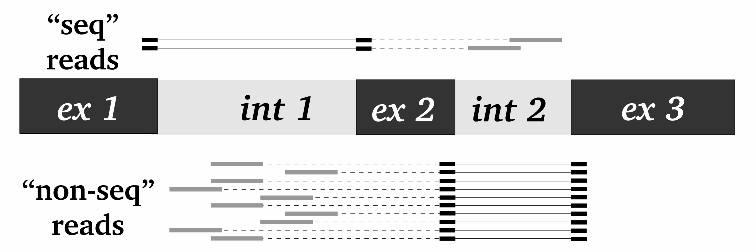

Figure S1: Read pairs supporting sequential ("seq") or non-sequential ("non-seq") splicing. Thick black lines represent ends that were split over a junction (and the thin black line connects the pieces from one end of a read pair). Thick gray lines represent ends mapped to the introns. Dashed line connects two ends of one read pair. In this example, number of "non-seq" read pairs equals 10 and the number of "seq" reads equals 2.

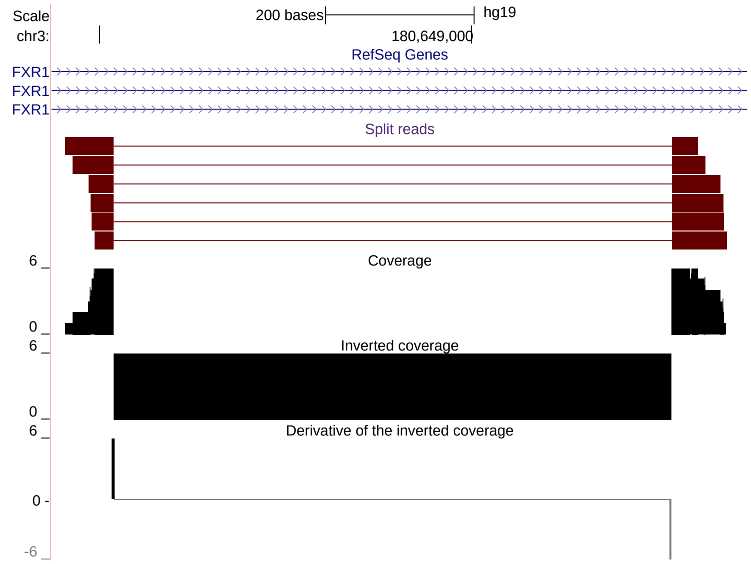

Figure S2: Schematic representation of the recursive splicing analysis. A split read (not mapped to an exon-exon junction) is used to calculate the inverted coverage. The derivative of the inverted coverage is then calculated, producing peaks at the positions where the split starts and drops at the positions where split ends. The size of peaks and drops equals the amount of reads split at this position.

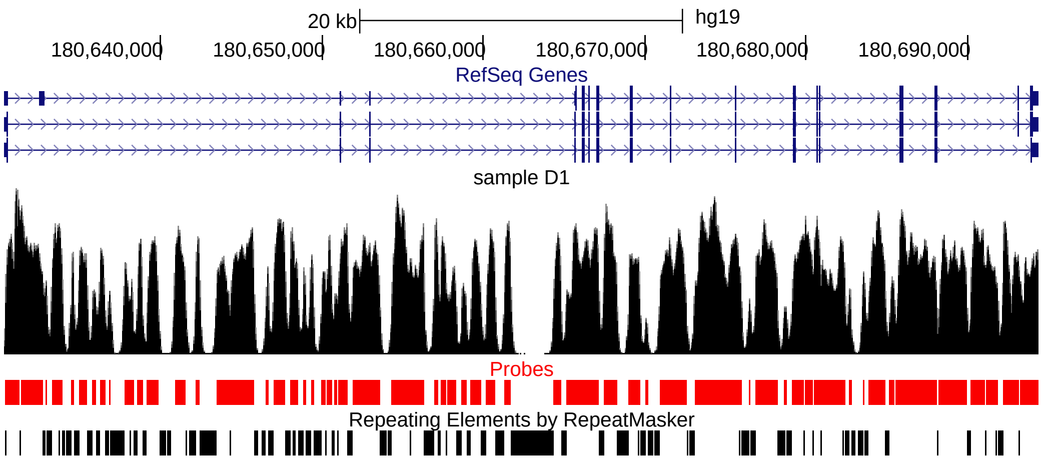

(a) Overview of the coverage across the whole gene being evenly distributed across exons and introns.

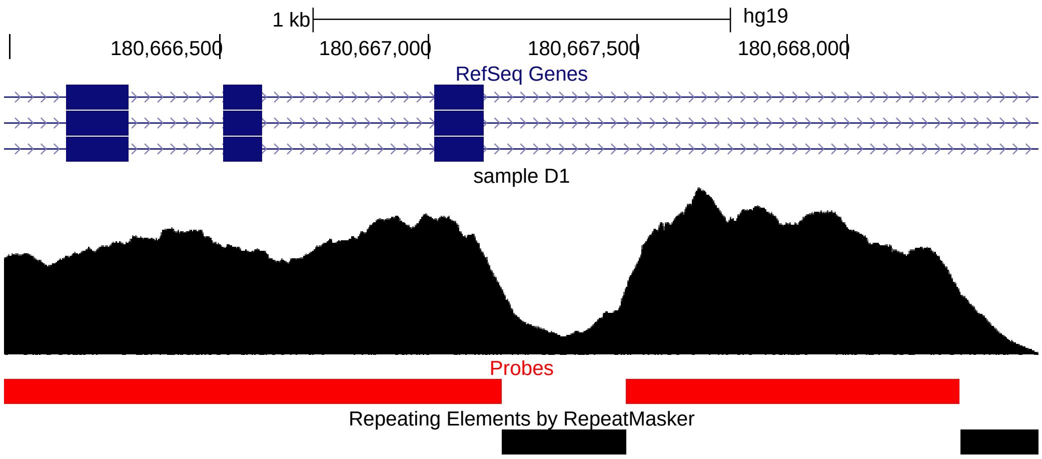

(b) Zoomed-in overview of the coverage, showing no difference between the coverage distribution across an exon and an intron.

Figure S3: Coverage of exons and introns in the DNA sample and its correlation with the probes and Repeat Masker regions. Top panel ("RefSeq Genes") indicates the NCBI annotation of the *FXR1* gene used for the analysis, thick blocks depicting exons and thin lines with arrows depicting introns. Second panel ("sample D1") shows the coverage of the DNA sample from the captured dataset (y-axis reflects the coverage, maximum coverage being over 2000). Third panel in red ("Probes") reflects the areas that have been covered by probes (blank areas depict the regions where no probes have been designed). The bottom panel ("Repeating Elements by Repeat Masker") indicate the Repeat Masker track provided by UCSC that has been used to design the probes (black areas depict repetitive elements that were not included in the probes).

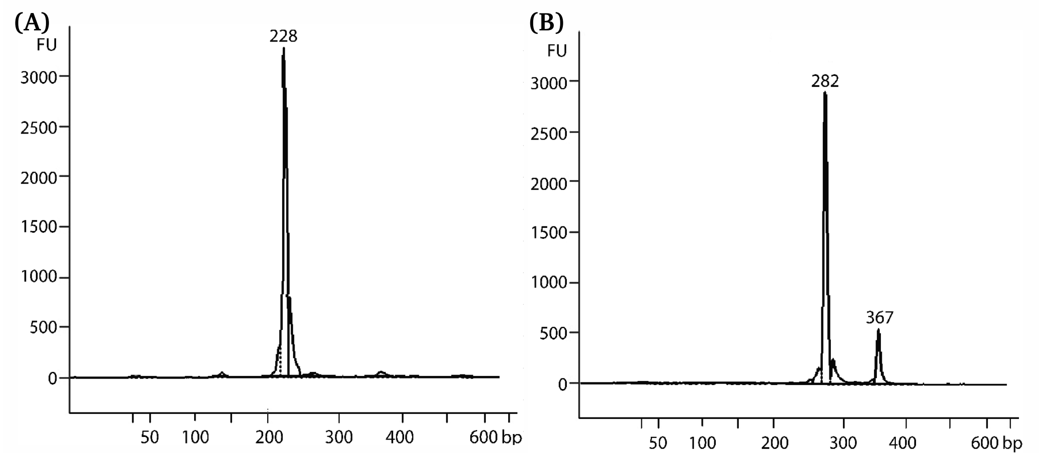

Figure S4: The results of PCR amplification experiments proving a skip of exon 2 and a retention of intron 13 in *FXR1*, as predicted *in silico* by the pipeline. (A) PCR primers were designed to anneal to exon 1 and exon 5, and this fragment was amplified. The highest peak indicates a fragment of exon 1-exon 5 without exon 2 (228 bp in length). The abundance of transcripts containing exon 2 is very low and the fragment containing exon 2 (571 bp) is not visible. (B) PCR primers were designed to anneal to exon 12 and exon 15, and the targeted fragments were amplified. The lower peak indicates a fragment with intron 13 inclusion (367 bp in length). The higher peak indicates a fragment without intron 13 (282 bp in length).

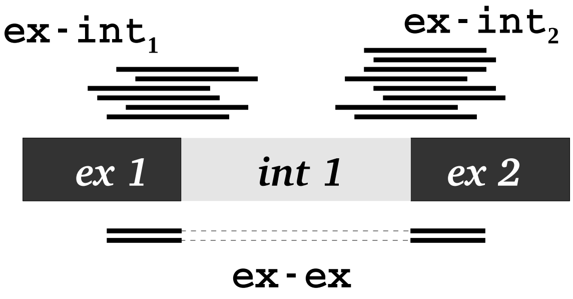

$$M = \frac{min(ex\text{-}int_1,\ ex\text{-}int_2)}{min(ex\text{-}int_1,\ ex\text{-}int_2) + ex\text{-}ex}$$

$$p\text{–}value = p\text{-}value_{binom}(ex\text{-}int_1,\ ex\text{-}int_2,\ 0.5) < 0.05$$

Figure S5: Calculating magnitude and likelihood for each intron of a gene in order to estimate its probability to be retained. *"M"* stands for "magnitude" and *"p-value"* stands for the *p*-value of the binomial test for likelihood.

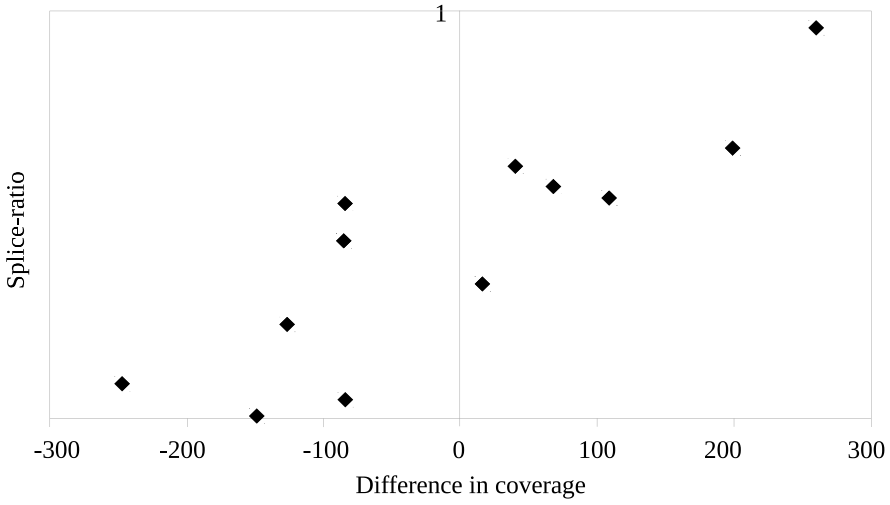

Figure S6: Linear correlation between the difference in median coverage ($\text{intron}_{i+1} - \text{intron}_i$) and the splice ratio. Correlation shown for the pre-mRNA N1 sample. Pearson correlation: -0.86. Spearman correlation: -0.84.

1	ATGGCGGAGC	TGACGGTGGA	GGTTCGCGGC	TCTAACGGGG	CTTTCTACAA	50
51	GGGATTTATC	AAAGATGTTC	ATGAAGACTC	CCTTACAGTT	GTTTTTGAAA	100
101	ATAATTGGCA	ACCAGAACGC	CAGGTTCCAT	TTAATGAAGT	TAGATTACCA	150
151	CCACCACCTG	ATATAAAAAA	AGAAATTAGT	GAAGGAGATG	AAGTAGAGGT	200
201	ATATTCAAGA	GCAAATGACC	AAGAGCCATG	TGGGTGGTGG	TTGGCTAAAG	250
251	TTCGGATGAT	GAAAGGAGAA	TTTTATGTCA	TTGAATATGC	TGCTTGTGAC	300
301	GCTACTTACA	ATGAAATAGT	CACATTTGAA	CGACTTCGGC	CTGTCAATCA	350
351	AAATAAAACT	GTCAAAAAAA	ATACCTTCTT	TAAATGCACA	GTGGATGTTC	400
401	CTGAGGATTT	GAGAGAGGCG	TGTGCTAATG	AAAATGCACA	TAAAGATTTT	450
451	AAGAAAGCAG	TAGGAGCATG	CAGAATTTTT	TACCATCCAG	AAACAACACA	500
501	GCTAATGATA	CTGTCTGCCA	GTGAAGCAAC	TGTGAAGAGA	GTAAACATCT	550
551	TAAGTGACAT	GCATTTGCGA	AGTATTCGTA	CGAAGTTGAT	GCTTATGTCC	600
601	AGAAATGAAG	AGGCCACTAA	GCATTTAGAA	TGCACAAAAC	AACTTGCAGC	650
651	AGCTTTTCAT	GAGGAATTTG	TTGTGAGAGA	AGATTTAATG	GGCCTGGCAA	700
701	TAGGAACACA	TGGTAGTAAC	ATCCAGCAAG	CTAGGAAGGT	TCCTGGAGTT	750
751	ACCGCCATTG	AGCTAGATGA	AGATACTGGA	ACATTCAGAA	TCTACGGAGA	800
801	GAGTGCTGAT	GCTGTAAAAA	AGGCTAGAGG	TTTCTTGGAA	TTTGTGGAGG	850
851	ATTTTATTCA	GGTTCCTAGG	AATCTCGTTG	GAAAAGTAAT	TGGAAAAAAT	900
901	GGCAAAGTTA	TTCAAGAAAT	AGTGGACAAA	TCTGGTGTGG	TTCGAGTGAG	950
951	AATTGAAGGG	GACAATGAAA	ATAAATTACC	CAGAGAAGAC	GGTATGGTTC	1000
1001	CATTTGTATT	TGTTGGCACT	AAAGAAAGCA	TTGGAAATGT	GCAGGTTCTT	1050
1051	CTAGAGTATC	ATATTGCCTA	TCTAAAGGAA	GTAGAACAGC	TAAGAATGGA	1100
1101	ACGCCTACAG	ATTGATGAAC	AGCTGCGACA	GATTGGTTCT	AGGTCTTATA	1150
1151	GCGGAAGAGG	CAGAGGTCGT	CGGGGACCTA	ATTACACCTC	CGGTTATGGT	1200
1201	ACAAATTCTG	AGCTGTCTAA	CCCCTCTGAA	ACGGAATCTG	AGCGTAAAGA	1250
1251	CGAGCTGAGT	GATTGGTCAT	TGGCAGGAGA	AGATGATCGA	GACAGCCGAC	1300
1301	ATCAGCGTGA	CAGCAGGAGA	CGCCCAGGAG	GAAGAGGCAG	AAGTGTTTCA	1350
1351	GGGGGTCGAG	GTCGTGGTGG	ACCACGTGGT	GGCAAATCCT	CCATCAGTTC	1400
1401	TGTGCTCAAA	GATCCAGACA	GCAATCCATA	CAGCTTACTT	GATAATACAG	1450
1451	AATCAGATCA	GACTGCAGAC	ACTGATGCCA	GCGAATCTCA	TCACAGTACT	1500
1501	AACCGTCGTA	GGCGGTCTCG	TAGACGAAGG	ACTGATGAAG	ATGCTGTTCT	1550
1551	GATGGATGGA	ATGACTGAAT	CTGATACAGC	TTCAGTTAAT	GAAAATGGGC	1600
1601	TAG**ATGATAG**	**TGAAAAAAAA**	**CCCCAGCGAC**	**GCAATCGTAG**	**CCGCAGGCGT**	1650
1651	**CGCTTCAGGG**	**GTCAGGCAGA**	**AGATAGACAG**	**CCAG**TCACAG	TTGCAGATTA	1700
1701	TATTTCTAGA	GCTGAGTCTC	AGAGCAGACA	AAGAAACCTC	CCAAGGGAAA	1750
1751	CTTTGGCTAA	AAACAAGAAA	GAAATGGCAA	AAGATGTGAT	TGAAGAGCAT	1800
1801	GGTCCTTCAG	AAAAGGCAAT	AAACGGCCCA	ACTAGTGCTT	CTGGCGATGA	1850
1851	CATTTCTAAG	CTACAGCGTA	CTCCAGGAGA	AGAAAAGATT	AATACCTTAA	1900
1901	AAGAAGAAAA	CACTCAAGAA	GCAGCAGTCC	TGAATGGTGT	TTCATAA	1947

Figure S7: Novel exon (in red) predicted by the pipeline and its location in the full-length *FXR1* transcript. The exon is located between exons 16 and 17 (according to the annotation used in this paper). The full-length transcript has been experimentally validated by Sanger sequencing.

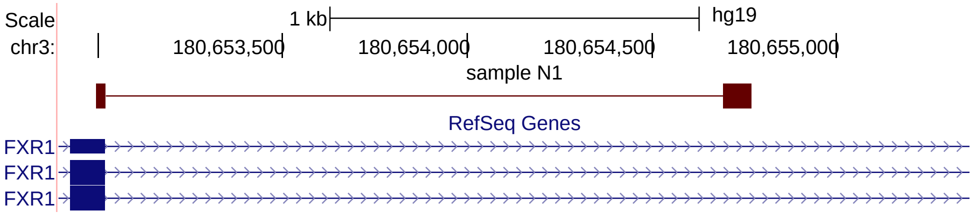

(a) Example of a recursive splicing event happening on the 5' end of intron 14.

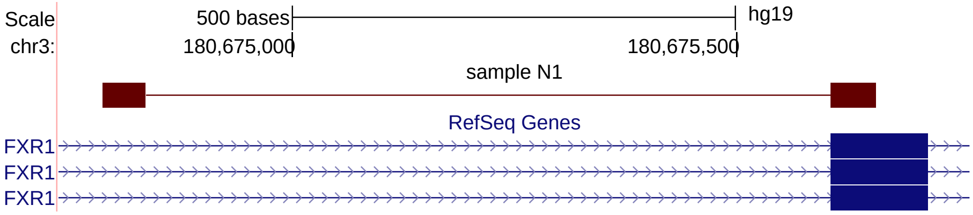

(b) Example of a recursive splicign event happening on the 3' end of intron 10.

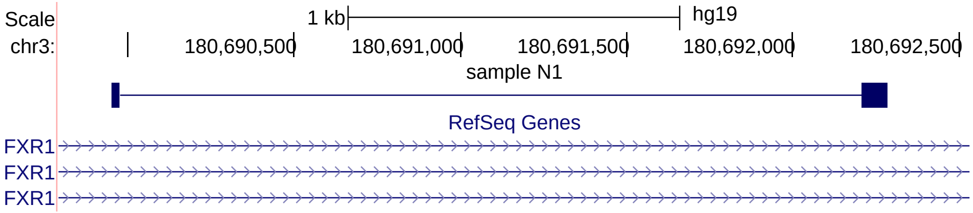

(c) Example of recursive splicing with two non-canonical splice sites in intron 16.

Figure S8: Examples of recursive splicing events found in the captured dataset. A black line in the middle represents one mapped read (*bam* file), thick part of the line represents aligned bases and thin part of the line represents the connection between the splitted reads. The bottom track on every panel (*wiggle* file) represents the positions of donor and acceptor splice sites. Blue arrows point at the donor splice sites and red arrows point at the acceptor splice sites.

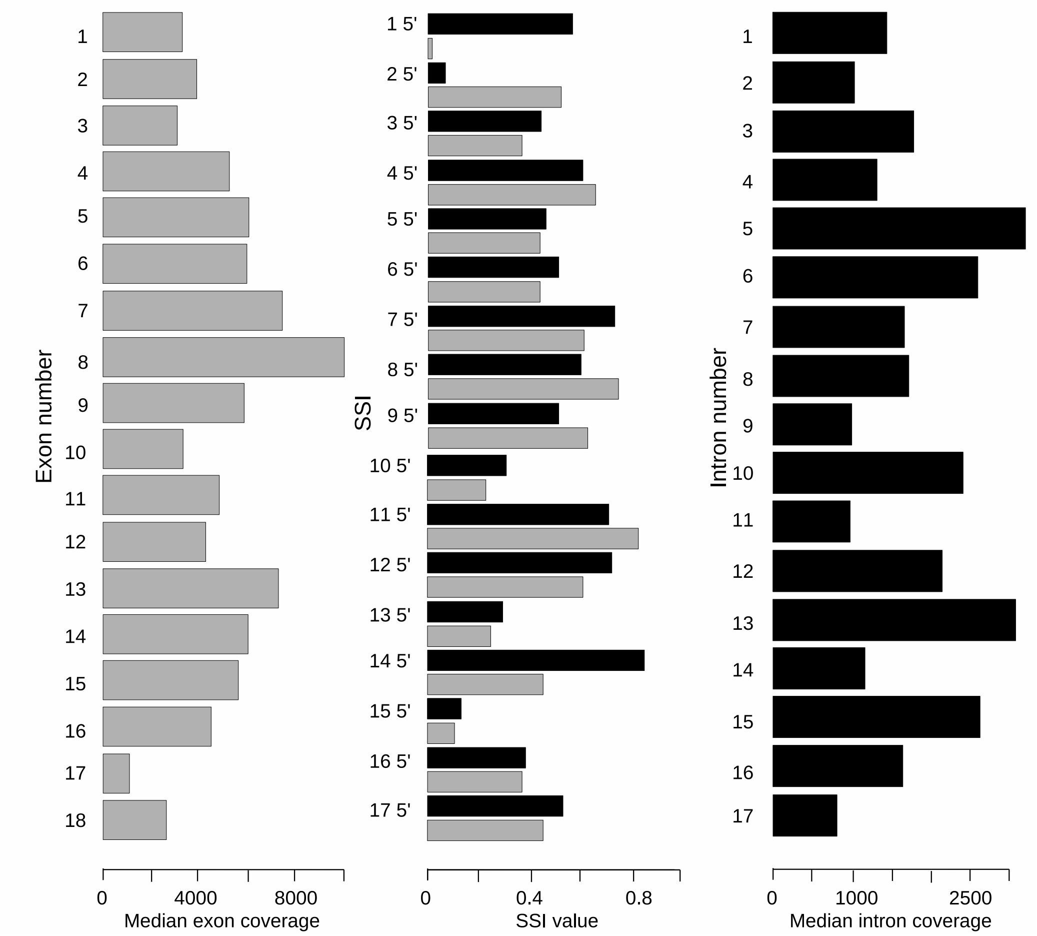

Figure S9: Splice site index (SSI) and medians of coverage of exons and introns in *FXR1* for sample C1 from the ENCODE dataset. Gray bars in the left panel represent the coverage of exons (exon 1 on top). Black bars in the middle panel represent SSI values for the 5' end of the introns and gray bars on the middle panel represent SSI values for the 3' end of the introns (intron 1 on top). Black bars in the right panel represent the coverage of introns (intron 1 on top). Data shown for chromatin RNA sample C2.

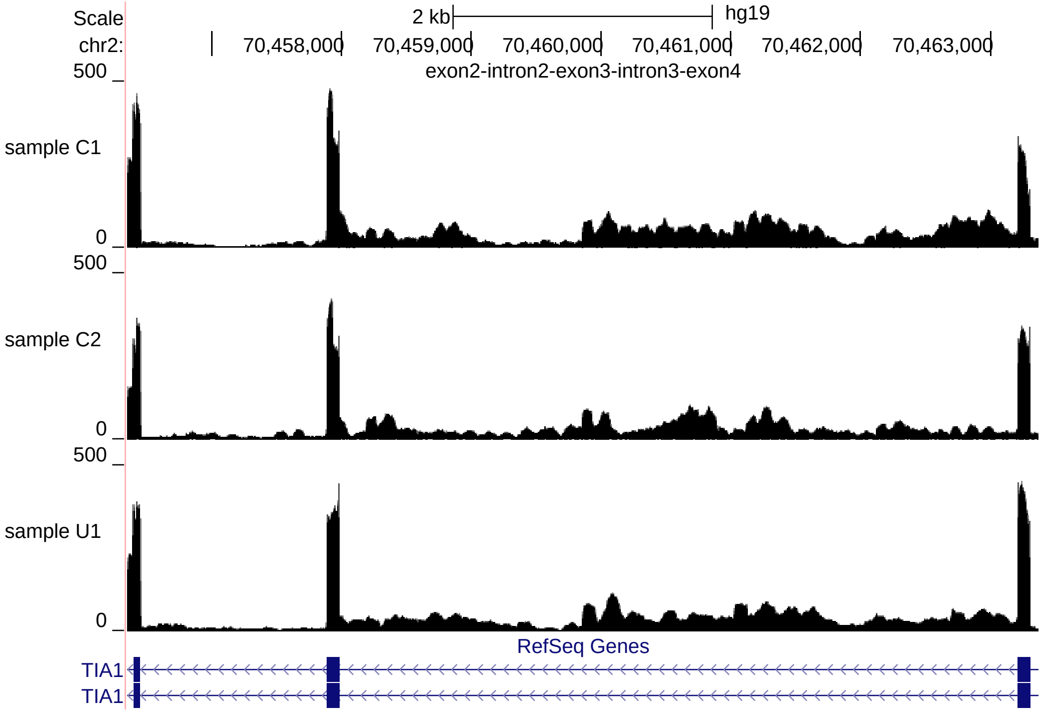

Figure S10: Graphical representation of two potentially non-sequentially spliced introns of *TIA1* – intron 2 is predicted to be spliced after intron 3. Top three panels represent the coverage from samples N1, N2 and U1. Coverage is the value on the y-axis, and the genomic coordinates are the value on the x-axis. The bottom panel represents the annotation of the gene available in the RefSeq database. Thick blocks represent exons, thin lines with arrows represent introns. Note that the gene is transcribed from the reverse strand and on the figure intron 2 is situated downstream (on the right) from intron 3. None of the introns with high coverage are annotated as retained introns, which gives an extra evidence that this is a case of non-sequential splicing and not a case of intron retention.
